# Excluding the ischiorectal fossa irradiation during neoadjuvant chemoradiotherapy with intensity-modulated radiotherapy followed by abdominoperineal resection decreases perineal complications in patients with lower rectal cancer

**DOI:** 10.1186/s13014-019-1338-5

**Published:** 2019-08-05

**Authors:** Maxiaowei Song, Jianhao Geng, Lin Wang, Yongheng Li, Xianggao Zhu, Xiaofan Li, Lan Mi, Aiwen Wu, Yifan Peng, Yunfeng Yao, Yangzi Zhang, Hongzhi Wang, Chen Shi, Yong Cai, Weihu Wang

**Affiliations:** 1 Key Laboratory of Carcinogenesis and Translational Research (Ministry of Education/Beijing), Department of Radiation Oncology, Peking University Cancer Hospital and Institute, Beijing, 100142 People’s Republic of China; 2 Key Laboratory of Carcinogenesis and Translational Research (Ministry of Education/Beijing), Department 3 of Gastrointestinal Surgery, Peking University Cancer Hospital and Institute, Beijing, 100142 People’s Republic of China; 3 Key Laboratory of Carcinogenesis and Translational Research (Ministry of Education/Beijing), Peking University Cancer Hospital and Institute, Beijing, 100142 People’s Republic of China

**Keywords:** Lower rectal cancer, Abdominoperineal resection, Ischiorectal fossa, Perineal wound complication, Intensity modulated radiotherapy

## Abstract

**Background:**

The aim of this study was to explore the impact of including or excluding the ischiorectal fossa (IRF) within the clinical target volume during neoadjuvant chemoradiotherapy (NCRT) using intensity modulated radiotherapy, in locally advanced lower rectal cancer (LALRC).

**Methods:**

We retrospectively analysed the data of 220 LALRC patients who received NCRT followed by abdominoperineal resection between January 2009 and January 2015. Six patients were excluded because of loss to follow-up, 90 patients received IRF irradiation (IRF group) while 124 patients did not (NIRF group). Survival, patterns of recurrence, and treatment toxicities were compared between the two groups.

**Results:**

Overall, patient/treatment variables were well balanced except for surgical technique. Perineal wound complications in the IRF and NIRF groups, were 40.0 and 24.2%, respectively (*p* = 0.010); corresponding 3-year perineal recurrence rates, local recurrence free survival, overall survival, and distant relapse free survival were 4.4% vs. 2.4% (*p* = 0.670), 88.1% vs. 95.0% (*p* = 0.079), 82.6% vs. 88.4% (*p* = 0.087), and 61.9% vs. 81.0% (*p* = 0.026), respectively. Multivariate analyses demonstrated the following factors to be significantly related to perineal wound complications: irradiation of the IRF (odds ratio [OR] 2.892, *p* = 0.002), anaemia (OR 3.776, *p* = 0.010), operation duration > 180 min (OR 2.486, *p* = 0.007), and interval between radiotherapy and surgery > 8 weeks (OR 2.400, *p* = 0.010).

**Conclusions:**

Exclusion of the IRF from the clinical target volume during NCRT using intensity-modulated radiotherapy in LALRC could lower the incidence of perineal wound complications after abdominoperineal resection, without compromising oncological outcomes.

**Electronic supplementary material:**

The online version of this article (10.1186/s13014-019-1338-5) contains supplementary material, which is available to authorized users.

## Background

Neoadjuvant chemoradiotherapy (NCRT) with abdominoperineal resection (APR) is the standard treatment for locally advanced lower rectal cancers (LALRC) unsuitable for sphincter-saving surgery. NCRT dose-fractionations are usually 50 Gy in 25 fractions or 50.4 Gy in 28 fractions. In our hospital, a 22-fraction intensity-modulated radiation therapy (IMRT) schedule, approved by the ethics committee, is used since 2007 [1–3]; it offers higher biological equivalent dose (BED), favourable down-staging, lower toxicities, and shorter treatment times.

Perineal wound complications in APR are high. NCRT increases risks of perineal wound complications [4–7] by 25 to 60% [8]. Data from a retrospective cohort in our hospital showed that the perineal complications were the most common events inducing great discomfort and inconvenience to patients [3].

The higher incidence of perineal wound complications, and low local recurrence rates after neoadjuvant treatment [1, 9, 10], have raised speculations that reducing perineal irradiation volumes, i.e. excluding IRF volumes, may reduce perineal wound complications [11, 12]. Since the levator ani muscles constitute an effective barrier against cancer spread into the IRF [11, 12] and nodal metastases in this area are rare, irradiation of the IRF has been considered unnecessary [11, 13].

However, confirmatory evidence from long-term follow-up studies with IRF-excluded clinical target volumes (CTVs) during NCRT is lacking. This retrospective study explored the impact of IRF-excluded CTVs on the incidence of perineal wound complications in patients with LALRC who underwent NCRT using IMRT, followed by APR; their impact on local recurrence was also assessed.

## Methods

### Patients

Data of 243 patients with LALRC who received NCRT using IMRT and standardized APR in a single-institutional database, between January 2009 and January 2015, were retrospectively reviewed. All patients had given informed consent before recruitment.

The inclusion criteria were: 1) histopathologically confirmed rectal adenocarcinoma, 2) clinical stage T3 to 4, or any stage T, and N+ tumours (7th ed. AJCC), determined by endorectal ultrasound or pelvic magnetic resonance imaging (MRI) and computed tomography (CT) (except patients in whom MRI was contraindicated), 3) candidates judged unsuitable for radical surgery by clinical examination and imaging, 4) APR with inferior tumour margins within 6 cm of the anal verge [14], 5) standard total mesorectal excision (TME), 6) no co-existing cerebrovascular or cardiac diseases.

The exclusion criteria were: 1) distant metastases, 2) history of pelvic radiation, 3) inflammatory bowel disease, 4) second malignancies, 5) cicatricial physique, 6) incomplete clinical or pathological data, 7) preoperative radiotherapy/chemotherapy or surgery in another hospital.

### Neoadjuvant chemoradiotherapy

CT-based simulation scans were taken with 5 mm slice-thickness, with patients in the supine position with a full bladder and a completely empty rectum. The scans extended from the fourth lumbar vertebra cranially to the mid-diaphyseal segments of the femur caudally. The gross tumour volume (GTV) was defined as the primary tumour and involved lymph nodes. The CTV was defined as the mesorectal and presacral regions, and obturator and internal iliac lymph nodes, with at least 2 cm margins from the superior and inferior extents of macroscopic disease in the rectum. The superior border of the pelvic CTV was the bifurcation of the common iliac artery. The inferior border was determined by tumour location, which was usually 2 to 3 cm below the GTV. Anatomically, the IRF commences where the inferior pudendal artery leaves the pelvis and enters the Alcock’s canal, and ends at the oblique plane joining the inferior level of the sphincter complex with the ischial tuberosity. For all patients treated between January 2009 and January 2013, IRF volumes were included in the CTV during delineation (IRF group), while the IRF was omitted from the CTV for all patients treated between January 2013 and January 2015 (NIRF group).

**Fig. 1:**
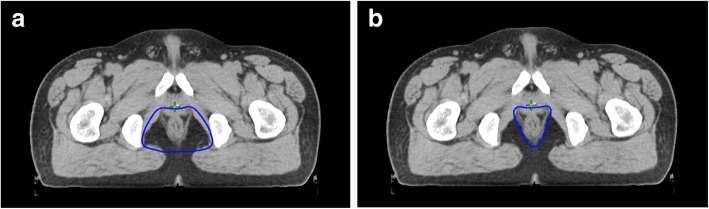
Delineation of the CTV in Ischiorectal Fossa area. Blue line: CTV; **a** Delineating the IRF area. **b** cT2–3: Omitting the IRF area

In the NIRF group, the CTV margins did not extend more than a few millimetres beyond the external sphincter or levator muscles in clinical stage T2 to T3 tumours. In clinical stage T4 tumours, the CTV margin of the diseased side extended at least 1 cm beyond the GTV (Fig. 1). The planning gross tumour volumes (PGTV) and planning target volumes (PTV) were determined by adding a 0.5 cm margin to the GTV and CTV, respectively [2].

All patients were treated with concomitant boost IMRT in a schedule of 22 fractions at 2.3 Gy and 1.9 Gy per fraction five times per week over 30 days for the PGTV and PTV, respectively. The total dose delivered to 95% PGTV and 95% PTV using 6/10 MV photons were 50.6 Gy and 41.8 Gy, respectively. All patients received concurrent chemotherapy with capecitabine (825 mg/m^2^ orally twice daily, 5 days/week) [2].

Organs at risk (OARs) included the urinary bladder, sigmoid, small bowel, and femoral head. All OARs were delineated to generate dose-volume histograms and maximum-tolerated doses and volumes. Dose reduction recommendations were in accordance with the protocol described in our previous report [2].

### Evaluation of toxicities and perineal wound complications

All patients were evaluated weekly for adverse events during NCRT, which were analysed according to the Common Terminology Criteria Adverse Events Version 4.0 (CTCAE v4.0). Patients were evaluated daily in hospital for perineal wound complications for at least the first week after APR. Additionally, follow-up data forms were completed in the outpatient clinic during follow-up visits to record any readmissions or complications after initial hospital discharge. All post-operative complications were recorded on examination by a surgeon.

Based on the surgeons’ clinical experience and evidence in the literature [6, 15, 16], perineal wound complications included: 1) perineal wound infections (erythematous tender swellings of the wound or surrounding tissues with purulent discharge) [17], 2) delayed healing (if the healing process exceeded 1 month after surgery) [17, 18], 3) perineal hernias (a bulge in the perineum associated with pain or discomfort, skin breakdown, intestinal obstruction, urinary symptoms, or pelvic evisceration) [19], 4) dehiscence (defined as separation of the skin at the perineal wound) [20]; 5) hematomas, seromas (defined as an abnormal collection of serous fluid in the deadspace without pus), [21] or haemorrhage; 6) fistulas or sinuses (perineal wound forming abnormal cavities or passages, which remains unhealed for > 6 months after surgery) [22]. The severity of postoperative perineal complications were graded according to the Clavien–Dindo classification system, which is applicable to a variety of surgical procedures [23].

### Surgical treatment and pathology procedures

In patients undergoing TME, surgery was performed approximately 6 to 12 weeks after completion of NCRT. Preoperative assessment included pelvic MRI/CT, and CT of the chest and abdomen [24]. In all patients, the extent of surgery was similar to extralevator abdominoperineal excisions (ELAPE), with cylindrical specimens [25]. Although both, laparoscopic-assisted and open resections were performed, the standard technique of perineal surgery was essentially similar among all patients.

Every surgical specimen underwent standardized pathological examination, including assessment of circumferential resection margins (CRM) [26]. Tumours were staged according to the seventh edition of the American Joint Committee on Cancer TNM classification [27].

### Study endpoints

The primary endpoints were incidence of perineal wound complications, and their severity as scored by the Clavien-Dindo classification system. The secondary endpoints included perineal recurrence, local recurrence free survival (LRFS), overall survival (OS) and distant relapse free survival. In patients who had undergone resection, perineal recurrence was defined as any detectable local disease at follow-up, occurring in the region of the anal sphincter complex and surrounding perianal and ischiorectal spaces [28]. LRFS was calculated from the date of initiation of radiotherapy to the date of local recurrence or death from any cause or last follow-up. OS was calculated from the date of initiation of radiotherapy to death from any cause, or last follow-up. Distant relapse free survival was calculated from the date of initiation of radiotherapy until the first event (distant relapse or death from any cause) or the last follow-up.

Patterns of acute toxicity and risk factors for perineal wound complications were also analysed.

### Follow-up

Follow-up visits included routine evaluation of symptoms, physical examination, gastrointestinal tumour markers, and blood tests. Abdominal ultrasonography or CT, pelvic CT/MRI, and chest CT were routinely performed every 3 months during the first 2 years, every 6 months for the next 3 years, and annually after 5 years.

### Statistical analysis

Categorical variables were compared using χ2 tests or the Fisher exact test; continuous data were compared between treatment groups using the Mann-Whitney *U* test. The Student’s t test was used to analyse differences in normally distributed data. LRFS, OS and distant relapse free survival were estimated using the Kaplan-Meier method. All factors with *p* < 0.1 were included in multivariate logistic regression analysis. Clinicopathologic variables were entered into a Cox proportional hazard multivariate model and analysed for effect on distant relapse free survival. Statistical analyses were performed using the SPSS 19.0 software package (IBM, Armonk, NY, United States); *p* values of < 0.05 were considered statistically significant.

## Results

### Patient characteristics

A total of 90 patients (42.1%) received IRF irradiation (IRF group) and 124 patients (57.9%) did not receive IRF radiation (NIRF group) between January 2009 and January 2013 and January 2013 and January 2015, respectively. All patients received the same radiotherapy dose, using the same technology, with standard concurrent capecitabine. Six patients were lost to follow-up and were excluded. Table 1 lists the patients’ and treatment characteristics. In the IRF group, the tumour invaded directly into the IRF in four patients. The tumour of one patient in the NIRF group invaded directly through the levators into the IRF.

**Table 1:** Baseline characteristics (*n* = 214)

Characteristic	*n* (%)		*p*
	IRF (*n* = 90)	NIRF (*n* = 124)	
Gender			
Male	63 (70.0)	88 (71.0)	0.878
Female	27 (30.0)	36 (29.0)	
Age (years)			
< 45	14 (15.5)	13 (10.5)	0.524
45–59	44 (48.9)	62 (50.0)	
≥ 60	32 (35.6)	49 (39.5)	
ECOG			
0	66 (73.3)	96 (77.4)	0.491
1	24 (26.7)	28 (22.6)	
BMI (kg/m^2^)			
< 18.5	3 (3.4)	4 (3.2)	0.245
< 25	51 (57.3)	57 (46.0)	
≥ 25	35(39.3)	63 (50.8)	
Distance from anal verge (cm)			
≤ 5	83 (92.2)	116 (93.5)	0.708
5–6	7 (7.8)	8 (6.5)	
c T Stage			
2	5 (5.6)	7 (5.7)	0.347
3	52 (57.8)	83 (66.9)	
4	33(36.6)	34(27.4)	
c N Stage			
0	9(10.0)	3(2.4)	0.056
1	9(10.0)	15(12.1)	
2	72(80.0)	106(85.5)	
Diabetes mellitus			
No	76 (84.4)	109 (87.9)	0.466
Yes	14 (15.6)	15 (12.1)	
Surgical technique			
Open surgery	67 (74.4)	73 (58.9)	0.018
Laparoscopic surgery	23 (25.6)	51 (41.1)	
Tumour histological type			
Well differentiated adenocarcinoma	8 (8.9)	15 (12.1)	0.059
Moderately differentiated adenocarcinoma	57 (63.3)	91 (73.4)	
Poorly differentiated adenocarcinoma, signet ring cell cancer or mucinous adenocarcinoma	16 (17.8)	8 (6.4)	
Uncertain differentiation type	9 (10.0)	10 (8.1)	
ypCR			
No	75 (83.3)	105 (84.7)	0.791
Yes	15 (16.7)	19 (15.3)	
ypN+			
No	69(76.7)	100 (80.6)	0.481
Yes	21 (23.3)	24 (19.4)	
CRM			
negative	89 (98.9)	123 (99.2)	1
positive	1 (1.1)	1 (0.8)	
Operation duration (min)			
≤ 180	48(53.3)	59(47.6)	0.406
> 180	42(46.7)	65(52.4)	
Time interval between NCRT and APR (weeks)			
≤ 8	47 (52.2)	51 (41.1)	0.108
> 8	43 (47.8)	73 (58.9)	

*Abbreviations*: *ECOG* Eastern Cooperative Oncology Group, *c* clinical, *pCR* Pathologic complete response, *NCRT* Neoadjuvant chemoradiotherapy, *BMI* Body mass index, *APR* Abdominoperineal resection, *CRM* Circumferential resection margins

### Treatment-related toxicity during chemoradiation

As shown in Additional file 1: Table S1, there were no treatment-related deaths in either group. Grade 3 toxicities included diarrhoea in four patients (1.9%), palmar-plantar erythrodysesthesia syndrome (PPES) (three patients, 1.4%), and myelosuppression in one patient (0.8%), who had leukocytopenia and thrombocytopenia. No differences in acute radiodermatitis were noted between the two groups (*p* = 0.688). One patient received sequential chemoradiotherapy due to impaired liver function. All 214 patients completed the planned radiotherapy schedule.

### Surgery

The median interval between NCRT and surgery was 7.9 weeks (range, 5.9 to 23 weeks) and 8.6 weeks (range, 5.7 to 18 weeks) in the IRF and NIRF groups, respectively. One patient in the IRF group initially achieved complete clinical response (cCR) managed by watch and wait but subsequently experienced local regrowth requiring radical surgery at 23 weeks after NCRT. The median duration of surgery was 177 min (range, 87 to 420 min) and 185.5 min (range, 75 to 374 min) in the IRF and NIRF groups, respectively. The median volumes of blood loss during surgery were 200 ml (range, 50 to 600 ml) and 100 ml (range, 30 to 800 ml) in the IRF and NIRF groups, respectively. Prophylactic antibiotics were usually prescribed within 24 h after surgery, and perineal wounds were managed with primary closure, allowing free drainage through or near the wound; vacuum suction or flap reconstruction was not performed [3].

### Adjuvant chemotherapy

Of the 214 patients who completed the planned surgery schedule, 153 (71.5%) received adjuvant chemotherapy (FOLFOX or CAPEOX or capecitabine), 53 (24.8%) underwent observation, and for 8 (3.7%), the therapy was unknown. In the IRF group, 65 (72.2%) patients received adjuvant chemotherapy while 19 (21.1%) patients did not, whereas in the NIRF group, 88 (71.0%) patients received adjuvant chemotherapy while 34 (27.4%) patients did not.

### Perineal wound complications

Perineal wound complications developed in 36 (40.0%) of the 90 patients and 30 (24.2%) of the 124 patients in the IRF group and NIRF groups, respectively (*p* = 0.01). The vast majority of perineal wound complications were of grade 1 severity. All severe complications (≥ grade 3) occurred in 4 (4.4%) patients in the IRF group; among them, 1 patient developed ischemic stroke after massive postoperative bleeding requiring emergency surgery under general anaesthesia for haemostasis, two patients underwent abscess debridement and drain replacements, 1 patient underwent bedside haemostasis under local anaesthesia using fixed-sutures to the resection surface with absorbable gauze. The various perineal wound complications are listed in Table 2. Wound infections and delayed wound healing were the most commonly reported complications. Among the total 5 patients who presented with tumour invading into the IRF, the four patients who presented perineal wound complications were all in the IRF group. Three patients experienced wound infection, and one developed haemorrhage. There were no severe complications.

**Table 2:** Types of perineal wound complications

Classification	*n* (%)	
	IRF group (*n* = 90)	NIRF group (*n* = 124)
Wound infection	21(23.3)	18(14.5)
Sinus and fistula	1(1.1)	3(2.4)
Wound dehiscence	1(1.1)	1(0.8)
Delayed wound healing	10(11.1)	4(3.2)
Wound hernia	0(0)	0(0)
Hematoma, seroma and haemorrhage	6(3.3)	5(4.0)
No. of patients	36(40.0)	30(24.2)
Number of adverse events	39(43.3)	31(25.0)

*Abbreviation*: *IRF* ischiorectal fossa irradiation, *NIRF* without ischiorectal fossa irradiation

4 patients had more than one complication

Univariate analysis demonstrated irradiation of IRF (*p* = 0.014), preoperative haemoglobin levels of ≤11 g/dL (*p* = 0.016), hypertension (*p* = 0.032), time interval between NCRT and APR > 8 weeks (*p* = 0.015), blood transfusions (*p* = 0.023), and operation duration > 180 min (*p* = 0.003) to be risk factors for perineal wound complications (Table 3). The odds ratios (OR) of the risk factors determined by multivariate analysis were: preoperative haemoglobin levels of ≤11 g/dL (*p* = 0.01, OR = 3.776), irradiation of IRF (*p* = 0.002, OR = 2.892), operation duration > 180 min (*p* = 0.007, OR = 2.486), time interval between NCRT and APR > 8 weeks (*p* = 0.01, OR = 2.400) (Table 4).

**Table 3:** Univariate logistic regression models for the risk of perineal wound complications

Variable	*n* (%)		OR (95%CI)	*p*
	Absent (*n* = 148)	Present (*n* = 66)		
Gender				
Male	102(68.9)	49(74.2)	Ref	
Female	46(31.1)	17(25.8)	0.431(0.401–1.477)	0.431
Age (years)				
< 45	18(12.2)	9(13.7)	Ref	
45–59	78(52.7)	28(42.4)	0.718(0.289–1.782)	0.475
≥60	52(35.1)	29(43.9)	1.115(0.444–2.799)	0.816
ECOG				
0	121(81.8)	48(72.7)	Ref	
1	27(18.2)	18(27.3)	1.681(0.848–3.330)	0.137
BMI (kg/m2)				
< 18.5	4(2.7)	3(4.5)	1.781(0.377–8.417)	0.466
< 25	76(51.7)	32(48.5)	Ref	
≥ 25	67(45.6)	31(47.0)	1.099(0.607–1.989)	0.755
ASA score				
I	14(9.4)	5(7.6)	Ref	
II	121(81.8)	52(78.8)	1.203(0.412–3.514)	0.735
III	13(8.8)	8(12.1)	1.723(0.447–6.636)	0.429
IV	0(0)	1(1.5)		1
p T Stage				
0–2	89(60.1)	40(60.6)	Ref	
3–4	59(39.9)	26(39.4)	0.981(0.542–1.775)	0.948
p N Stage				
0	117(79.1)	52(78.8)	Ref	
1–2	31(20.9)	14(21.2)	1.016(0.499–2.068)	0.965
Diabetes mellitus				
No	130(87.8)	55(83.3)	Ref	
Yes	18(12.2)	11(16.7)	0.640–3.259	0.376
Hypertension				
No	117(79.1)	43(65.2)	Ref	
Yes	31(20.9)	23(34.8)	2.019(1.062–3.839)	0.032
Preoperative albumin level (g/dl)				
≥ 35	137(97.9)	61(96.8)	Ref	
< 35	3(2.1)	2(3.2)	1.497(0.244–9.190)	0.663
Preoperative haemoglobin level (g/dl)				
≥ 11	137(93.8)	53(82.8)	Ref	
< 11	9(6.2)	11(17.2)	3.159(1.239–8.058)	0.016
Surgical technique				
Laparoscopic surgery	50(33.8)	24(36.4)	Ref	
Open surgery	98(66.2)	42(63.6)	0.893(0.487–1.637)	0.714
Smoking				
No	78(52.7)	27(40.9)	Ref	
Yes	70(47.3)	39(59.1)	1.610(0.895–2.896)	0.112
Bleeding volume (ml)				
≤ 200	123(84.2)	51(77.3)	Ref	
> 200	23(15.8)	15(22.7)	1.573(0.760–3.257)	0.223
Blood transfusion				
No	137(92.6)	54(81.8)	Ref	
Yes	11(7.4)	12(18.2)	2.768(1.152–6.650)	0.023
Operation duration (min)				
≤ 180	84(56.8)	23(34.8)	Ref	
> 180	64(43.2)	43(65.2)	2.454(1.344–4.479)	0.003
Time interval between NCRT and APR				
≤ 8 weeks	76(51.4)	22(33.3)	Ref	
> 8 weeks	72(48.6)	44(66.7)	2.111(1.153–3.865)	0.015
Irradiation of IRF				
No	94(63.5)	30(45.5)	Ref	
Yes	54(36.5)	36(54.5)	2.089(1.159–3.764)	0.014

*Abbreviations*: *ECOG* Eastern Cooperative Oncology Group, *p* pathological, *NCRT* Neoadjuvant chemoradiotherapy, *BMI* Body mass index, *APR* Abdominoperineal resection, *IRF* Ischiorectal fossa, *ASA* American Society of Anesthesiologists, *Ref* Reference

**Table 4:** Multivariate logistic regression models for the risk of perineal wound complications

Variable	*n* (%)		OR(95%CI)	*p*
	Absent (*n* = 148)	Present (*n* = 66)		
Preoperative haemoglobin level (g/dl)				
≥ 11	137(93.8)	53(82.8)	Ref	
< 11	9(6.2)	11(17.2)	3.776 (1.380–10.355)	0.010
Operation duration (min)				
≤ 180	84(56.8)	23(34.8)	Ref	
> 180	64(43.2)	43(65.2)	2.486 (1.287–4.804)	0.007
Hypertension				
No	117(79.1)	43(65.2)	Ref	
Yes	31(20.9)	23(34.8)	1.962 (0.958–4.019)	0.065
Blood transfusion				
No	137(92.6)	54(81.8)	Ref	
Yes	11(7.4)	12(18.2)	1.702 (0.577–5.022)	0.336
Time interval between NCRT and APR (weeks)				
≤ 8	76(51.4)	22(33.3)	Ref	
> 8	72(48.6)	44(66.7)	2.400 (1.237–4.656)	0.010
Irradiation of IRF				
No	94(63.5)	30(45.5)	Ref	
Yes	54(36.5)	36(54.5)	2.892 (1.490–5.615)	0.002

*Abbreviations*: *NCRT* Neoadjuvant chemoradiotherapy, *APR* Abdominoperineal resection, *IRF* Ischiorectal fossa, *Ref* Reference

### Survival analysis

The median duration of follow-up was 40.7 months (range 9.1 to 60.7 months). None of the perineal recurrences occurred in patients with IRF invasion in the two groups. So the local perineal recurrence rates did not differ significantly between the groups (*p* = 0.67), with recurrences in four (4.4%) and three patients (2.4%) in the IRF group and NIRF groups, respectively.

**Fig. 2:**
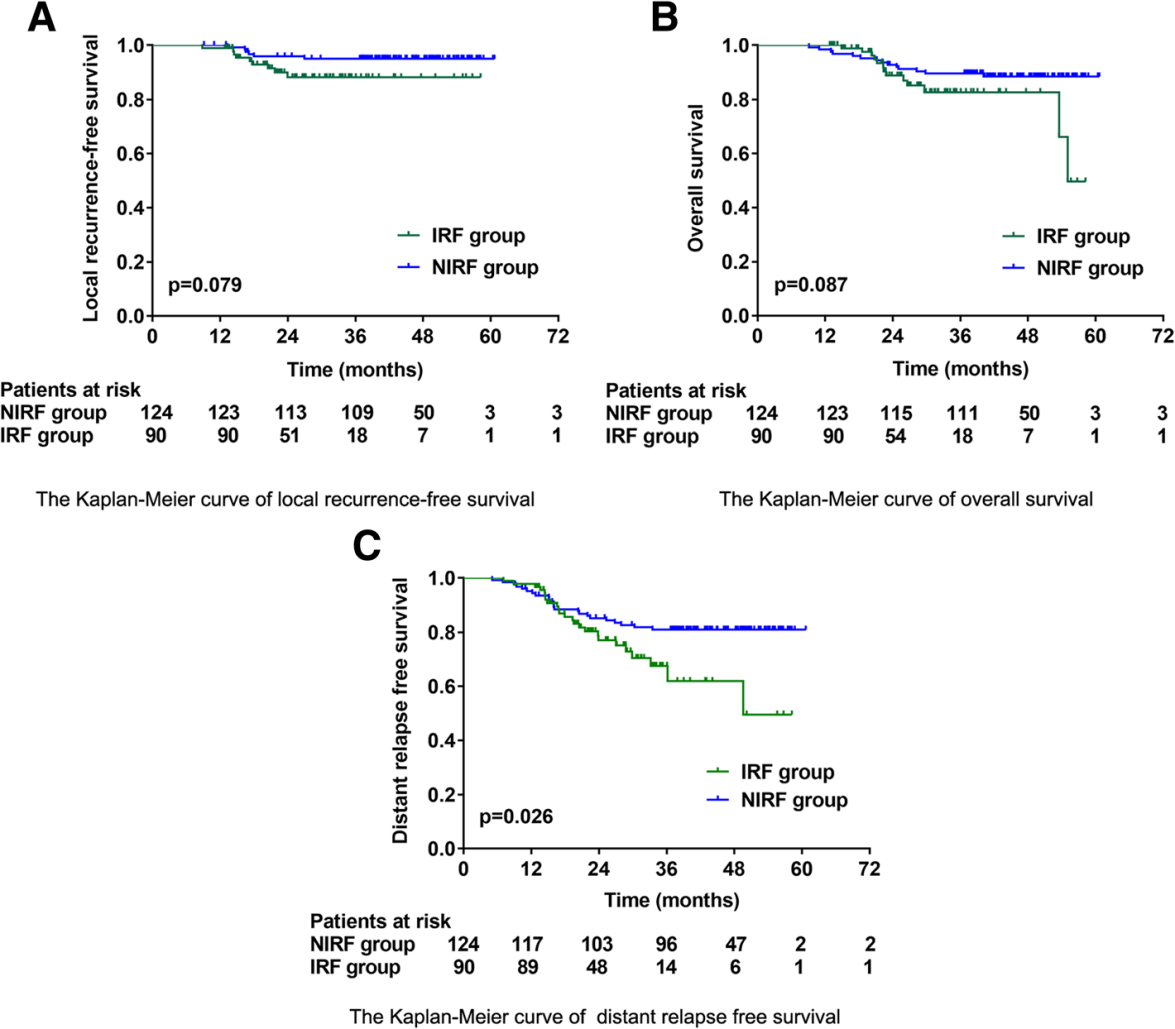
Survival analysis. **a, b, c** Kaplan-Meier estimation of local recurrence free survival (LRFS), overall survival (OS) and distant relapse free survival. *Abbreviation:* IRF = ischiorectal fossa irradiation; NIRF = without ischiorectal fossa irradiation

LRFS and OS did not differ significantly between the two groups; the estimated 3-year LRFS was 88.1% with 95% confidence interval (CI) of 80.7 to 95.5% in the IRF group vs. 95.0% (95% CI, 91.1 to 98.9%) in the NIRF group, log rank *p* = 0.079; the estimated 3-year OS was 82.6% (95% CI, 73.0 to 92.2%) in the IRF group vs. 88.4% (95% CI, 82.7 to 94.1%) in the NIRF group, log rank *p* = 0.087. For the distant relapse free survival, we detected a significant difference between the two groups. The estimated 3-year distant relapse free survival was 61.9% (95% CI, 46.6 to 77.2%) in the IRF group vs. 81.0% (95% CI, 73.9 to 88.1%) in the NIRF group, log rank *p* = 0.026 (Fig. 2).

For distant relapse free survival, the results of the univariate and multivariate analyses of prognostic factors were summarised in Additional file 1: Table S2. The independent prognostic factors for distant relapse free survival were occurrence of perineal wound complications (hazard ratio [HR] 1.89, 95% CI 1.05 to 3.41), pathological T3 stage (HR 6.66, 95% CI 1.57 to 28.19), pathological T4 stage (HR 21.55, 95% CI 3.57 to 130.05), and CRM positive (HR 16.03, 95% CI 3.39 to 75.95).

## Discussion

In the precision-therapy era, planning ideal irradiation fields based on rectal tumour location is a considerable challenge to the clinician. In LALRC, the inclusion of entire IRF volumes into the CTV remains controversial [11, 12, 28–30]. Radiotherapy technology has advanced rapidly over the past three decades, from two- to three-dimensional conformal radiotherapy to high precision intensity-modulated radiation therapy (IMRT). In the two-dimensional radiotherapy era, square irradiation fields covering the entire lateral lymph node areas, lower tumour boundaries, and perineal scars could not spare the IRF in patients with LALRC. Precision radiotherapy, however, allows optimal conformity and precise radiation delivery to tumours. It is therefore essential to investigate the controversies regarding irradiation of the IRF to allow normal tissue sparing. The findings from our study demonstrate that IRF-excluded target volumes in LALRC could decrease perineal complications after APR, with similar local recurrence and overall survival compared with conventional IRF-included target volumes. Further, the occurrence of perineal wound complications might be associated with decreased distant relapse free survival due to immunosuppression and adjuvant treatment delay or omission. To the best of our knowledge, this study was the first to compare the incidence of perineal complications in LALRC after APR following NCRT using IMRT, with either IRF- inclusive or exclusive volumes.

The IRF is a triangular area bounded by the levator ani muscles, the obturator and gluteal muscles, and the ischial tuberosity [12]. This area is partitioned by a thin horizontal fascia into the perianal and ischiorectal spaces, which contain adipose tissue, pudendal nerve branches, superficial branches of the internal pudendal vessels, and lymphatic trunks [31, 32]. Additionally, the ischiorectal fat and anal canal below the pectinate line, developmentally derived from the ectoderm, constitutes an effective barrier against cancer. In theory, advanced lower rectal cancer could spread to the surrounding anorectal tissues and metastasize to lymph nodes in this area. Some researchers even proposed that recurrences in perineal region likely originated from implantation during surgery [11]. However, previous studies demonstrated that infiltration or nodal metastases to the ischiorectal space are rare in rectal cancer [13, 33, 34], occurring in approximately 2% [35]. Heald et al. found that the lymphatic drainage of the rectum did not extend below the levator muscles. They observed that pathologically, most nodes outside the mesorectal “package” did not contain cancer cells [36].

The inferior pelvic subsite (IPS), consisting of the anal sphincter complex and surrounding perianal and ischiorectal spaces is one of five predominant areas at risk of local recurrence. In a previous study, overall recurrence in the IPS was approximately 4% (53/1188), which increased to 8% (18/234) for tumours located < 6 cm from the anal margin [28]. Since the IPS is at particular risk of local recurrence in patients with tumours < 6 cm from the anal margin, and in those undergoing APR, researchers initially proposed inclusion of this area in the CTV. However, most of the available data on recurrence came from retrospective analyses before the 1980’s, when surgical techniques were relatively primitive [28, 37]. Furthermore, these studies reported IPS recurrences without considering IRF-specific recurrences separately; failures in the IRF are very rare in the absence of invasion [30].

In practice, the IRF may be excluded from the CTV owing to lower incidences of invasion, metastases, and recurrence. Neoadjuvant radiotherapy significantly increases perineal wound problems after APR [16]. Marijnen et al. demonstrated that patients treated with perineum-included irradiation fields had higher rates of local wound complications compared to patients with perineum-excluded fields (31% vs. 18%) [7]. The adverse effects of preoperative radiation therapy directly relate to normal tissue injury through progressive occlusive vasculitis and fibrosis [38]. Consequently, we speculated that IRF-excluded CTVs would reduce the occurrence of perineal wound complications after APR. In 2016, the European Working Group (EWG) proposed to include the IRF into the CTV when the tumour invaded the external anal sphincter. In patients undergoing APR for tumours superficially infiltrating the IRF, the EWG suggested omitting the IRF during preoperative radiotherapy [12]. In our study, reducing irradiation volumes of the perineum clearly lowered the incidence of perineal wound complications. Further investigation revealed IRF irradiation to be a significant risk factor for perineal wound complications. From the radiation biology perspective, these results could indicate radiation induced collateral normal tissue damage [15, 38].

Perineal wound complications are multifactorial. Traditionally, preoperative radiation, high body mass index (BMI), poor nutrition, diabetes mellitus, and certain types of wound closure were considered risk factors for perineal wound complications [4]. Additionally, surgical factors including prolonged operation duration and massive bleeding increase the risk of wound infections [5]. There is no evidence on whether laparoscopic surgery reduces postoperative perineal wound complications [39, 40]. Although sample size was limited, our data indicates that factors including preoperative anaemia, operation duration > 180 min, and time intervals between NCRT and APR > 8 weeks are also risk factors for wound complications.

Our study shows a significant association between perineal wound complications and distant relapse free survival, which might indicate that postoperative complications could be related to early distant recurrence. The precise mechanism of this association remains to be determined. Some authors suggested that it is due to immunosuppression and adjuvant treatment delay [41, 42]. Given the limitations of our study, all these questions need to be researched and answered in the next step.

A search of literature does not reveal any long-term follow-up data from IRF-excluded NCRT cohorts. Our follow-up data revealed that in the absence of cT4 tumours with massive infiltration, smaller perineal irradiation volumes do not increase perineal recurrences or worsen LRFS. We therefore suggest excluding the IRF during NCRT with IMRT in patients undergoing subsequent APR for lower rectal cancers. Further, findings of this study might not apply currently to patients receiving postoperative radiotherapy (RT) after APR.

The present study has several limitations. Firstly, it was a retrospective cohort study, with historical case-controls and was based on a single centre, introducing selection bias and limiting the availability of variables. Additionally, the historical control (IRF group) and NIRF groups differed in time scale. Secondly, perineal complications were multifactorial, making well-controlled comparisons of any variable difficult. Thirdly, the duration of follow-up was relatively short; longer follow-up is necessary to assess recurrence and survival. Nevertheless, this study included a relatively large sample size from a single dedicated cancer centre treated with contemporary NCRT with IMRT, and surgery. A future prospective randomized clinical study will be designed to explore the efficacy and toxicity of IRF-excluding neoadjuvant radiotherapy.

## Conclusions

IRF-excluded CTVs during neoadjuvant chemoradiotherapy using IMRT potentially reduce perineal wound complications after APR in LALRC, without affecting local recurrence or overall survival. Further confirmation by prospective studies is warranted.

## Additional file

Additional file 1: **Table S1.** Treatment-related toxicity during chemoradiation. **Table S2.** Univariate and multivariate Cox proportional hazards model for distant relapse free survival. (DOCX 24 kb).

## Data Availability

The datasets used and/or analysed during the current study are available from the corresponding author on reasonable request.

## Abbreviations

APR: Abdominoperineal resection
BED: Biological equivalent dose
BMI: Body mass index
cCr: Complete clinical response
CI: Confidence interval
CRM: Circumferential resection margins
CT: Computed tomography
CTCAE v4.0: Common Terminology Criteria Adverse Events Version 4.0
CTV: Clinical target volume
ELAPE: Extralevator abdominoperineal excisions
EWG: European Working Group
GTV: Gross tumour volume
HR: Hazard ratio
IMRT: Intensity-modulated radiotherapy
IPS: Inferior pelvic subsite
IRF: Ischiorectal fossa
LALRC: Locally advanced lower rectal cancer
LRFS: Local recurrence free survival
MRI: Magnetic resonance imaging
NCRT: Neoadjuvant chemoradiotherapy
OAR: Organs at risk
OR: Odds ratio
OS: Overall survival
PGTV: Planning gross tumour volumes
PPES: Palmar-plantar erythrodysesthesia syndrome
PTV: Planning target volumes
RT: Radiotherapy
TME: Total mesorectal excision
