# Supplementary material for: Excluding the ischiorectal fossa irradiation during neoadjuvant chemoradiotherapy with intensity-modulated radiotherapy followed by abdominoperineal resection decreases perineal complications in patients with lower rectal cancer
Source: Radiat Oncol. 2019 Aug 5;14:138. doi: 10.1186/s13014-019-1338-5 (PMC6683419; doi:10.1186/s13014-019-1338-5)
Supplement: Supplementary file 1 — Table S1. Treatment-related toxicity during chemoradiation. Table S2. Univariate and multivariate Cox proportional hazards model for distant relapse free survival. (DOCX 24 kb). [file 13014_2019_1338_MOESM1_ESM.docx]

**Additional file 1: Table S1** Treatment-related toxicity during chemoradiation

| Toxicity | n (%) | | | | | | | | p |
| --- | --- | --- | --- | --- | --- | --- | --- | --- | --- |
|  | IRF group (n=90) | | | | NIRF group (n=124) | | | |  |
|  | Grade 1 | Grade 2 | Grade 3 | Grade 4 | Grade 1 | Grade 2 | Grade 3 | Grade 4 |  |
| Myelosuppression | 29(32.2) | 9(10.0) | 0 | 0 | 53(42.7) | 14(11.3) | 1(0.8) | 0 | 0.138 |
| Nausea | 22(24.5) | 0 | 0 | 0 | 34(27.6) | 1(0.8) | 0 | 0 | 0.058 |
| Acute Radiodermatitis | 51(57.3) | 7(7.9) | 0 | 0 | 72(58.5) | 11(8.9) | 0 | 0 | 0.688 |
| Diarrhea | 35(39.8) | 19(21.6) | 1(1.1) | 0 | 54(43.9) | 23(18.7) | 3(2.4) | 0 | 0.865 |
| PPES | 14(16.1) | 7(8.0) | 1(1.1) | 0 | 22(17.7) | 6(4.8) | 2(1.6) | 0 | 0.791 |
| Liver function damage | 3(3.5) | 3(3.5) | 0 | 0 | 14(11.3) | 5(4.0) | 0 | 0 | 0.076 |

Abbreviations: PPES= Palmar-plantar erythrodysesthesia syndrome

**Additional file 1: Table S2** Univariate and multivariate Cox proportional hazards model for distant relapse free survival.

| Variable | Univariate | | Multivariate | |
| --- | --- | --- | --- | --- |
|  | HR (95% CI) | p | HR (95% CI) | p |
| Gender | 1.068(0.578-1.973) | 0.834 |  |  |
| Age (years) |  |  |  |  |
| <45 | 1 |  |  |  |
| 45-59 | 0.967(0.424-2.202) | 0.936 |  |  |
| ≥60 | 1.256(0.477-3.303) | 0.644 |  |  |
| ECOG (1 vs. 0) | 1.698(0.928-3.107) | 0.086 | 1.553(0.805-2.995) | 0.189 |
| BMI (kg/m2) |  |  |  |  |
| <18.5 | 0.470(0.064-3.459) | 0.459 |  |  |
| <25 | 1 |  |  |  |
| ≥25 | 0.661(0.366-1.196) | 0.171 |  |  |
| Tumor histological type |  |  |  |  |
| Well differentiated adenocarcinoma | 1 |  |  |  |
| Moderately differentiated adenocarcinoma | 1.240(0.486-3.167) | 0.652 |  |  |
| Poorly differentiated adenocarcinoma, signet ring cell cancer or mucinous adenocarcinoma | 1.311(0.379-4.537) | 0.669 |  |  |
| Uncertain differentiation type | 0.532(0.103-2.741) | 0.450 |  |  |
| p T Stage |  |  |  |  |
| 0 | 1 |  |  |  |
| 1 | 4.252(0.599-30.198) | 0.148 | 2.949(0.405-21.452) | 0.286 |
| 2 | 2.241(0.491-10.228) | 0.298 | 2.081(0.456-9.506) | 0.344 |
| 3 | 8.340(1.992-34.908) | 0.004 | 6.659(1.573-28.193) | 0.010 |
| 4 | 22.989(3.823-138.241) | 0.001 | 21.550(3.571-130.047) | 0.001 |
| p N Stage(N+ vs. N0) | 2.208(1.207-4.039) | 0.010 | 1.855(0.989-3.482) | 0.054 |
| pCR(positive vs. negative) | 0.108(0.015-0.728) | 0.028 | 0.198(0.011-3.440) | 0.267 |
| CRM(positive vs. negative) | 21.725(4.860-97.109) | < 0.001 | 16.033(3.385-75.949) | < 0.001 |
| Lymphovascular invasion(positive vs. negative) | 4.281(1.326-13.827) | 0.015 | 2.587(0.748-8.950) | 0.133 |
| Perineural invasion(positive vs. negative) | 1.818(0.441-7.494) | 0.408 |  |  |
| Adjuvant chemotherapy (Yes vs. No) | 1.282(0.594-2.765) | 0.527 |  |  |
| Time interval between NCRT and APR(>8 weeks vs. ≤8 weeks) | 0.834(0.471-1.479) | 0.535 |  |  |
| Perineal wound complication (Yes vs. No) | 1.854(1.039-3.308) | 0.037 | 1.893(1.052-3.405) | 0.033 |

Abbreviations: ECOG= Eastern Cooperative Oncology Group; p=pathological; pCR=Pathologic complete response; NCRT=Neoadjuvant chemoradiotherapy; BMI= Body mass index; APR= Abdominoperineal resection; CRM= Circumferential resection margins;
